# Supplementary figures and images for: Molecular Interplay between Non-Host Resistance, Pathogens and Basal Immunity as a Background for Fatal Yellowing in Oil Palm (Elaeis guineensis Jacq.) Plants
Source: Int J Mol Sci. 2023 Aug 18;24(16):12918. doi: 10.3390/ijms241612918 (PMC10454536; doi:10.3390/ijms241612918)

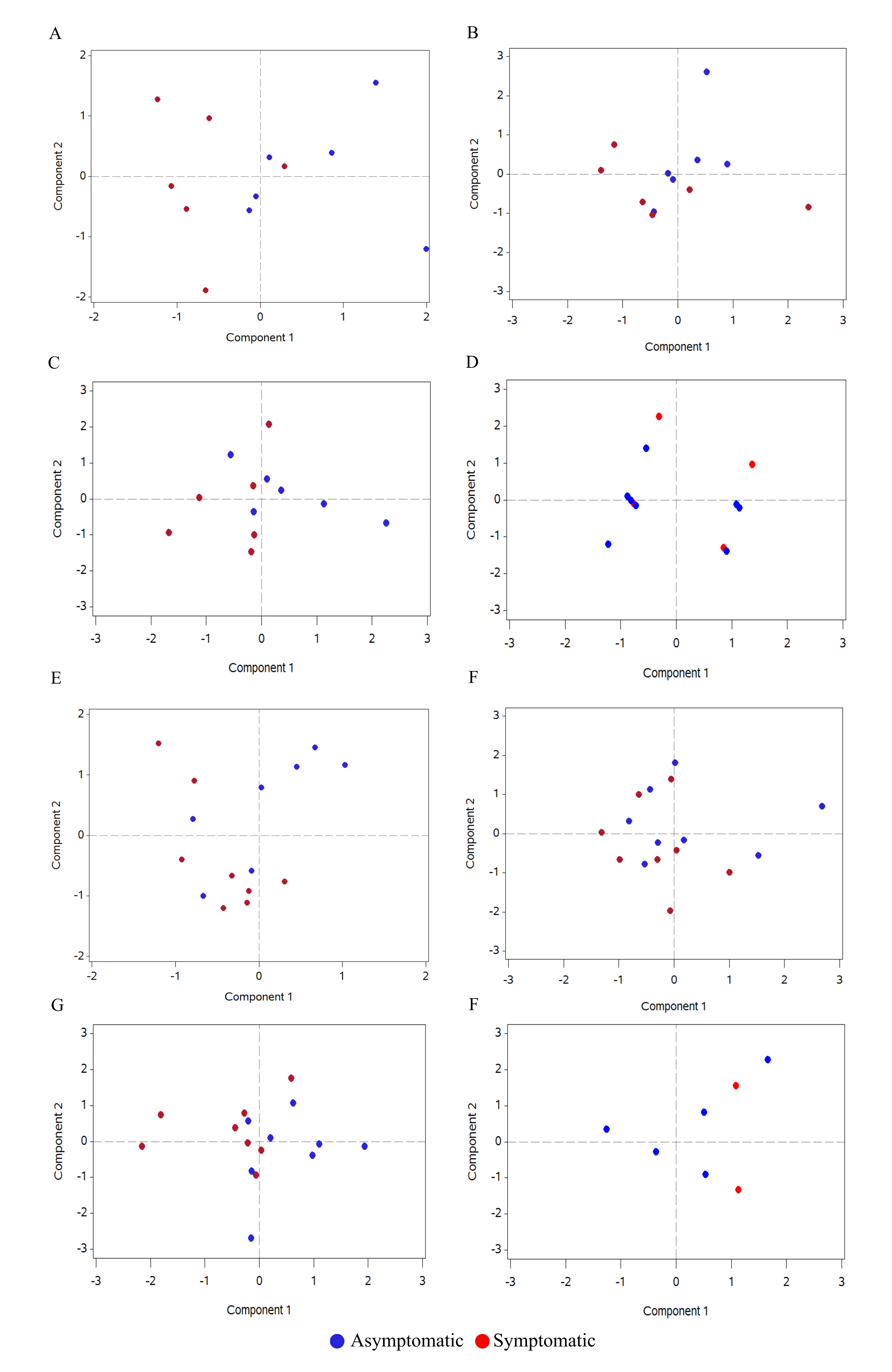

Supplement: Supplementary file 1 [file ijms-24-12918-s001.zip › Bittencourt et al_Figure S01.jpg]

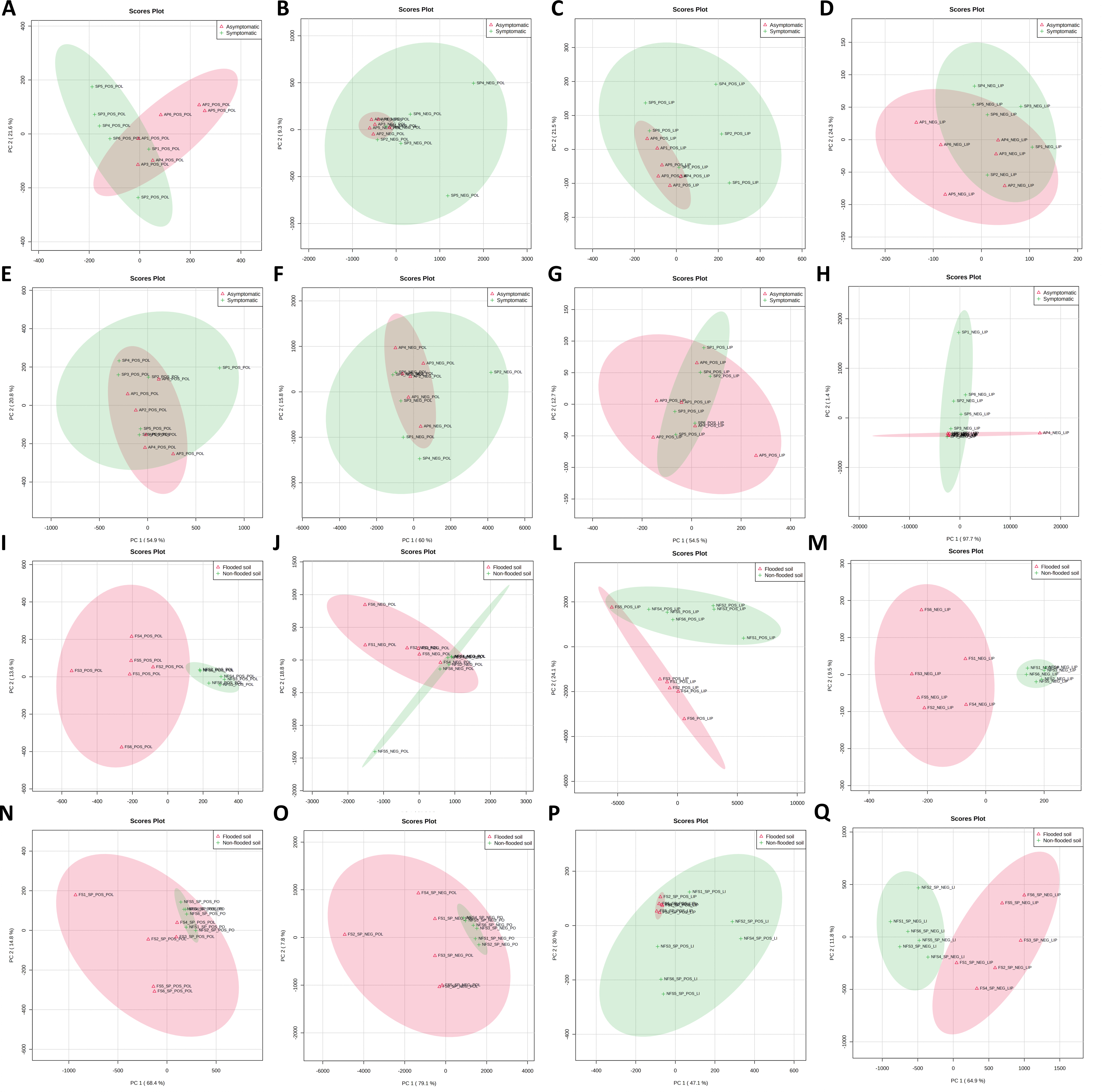

Supplement: Supplementary file 1 [file ijms-24-12918-s001.zip › Bittencourt et al_Figure S02.jpg]
